# The reputational and ethical consequences of deceptive chatbot use

**DOI:** 10.1038/s41598-023-41692-3

**Published:** 2023-09-27

**Authors:** Jack McGuire, David De Cremer, Yorck Hesselbarth, Leander De Schutter, Ke Michael Mai, Alain Van Hiel

**Affiliations:** 1 Department of Management and Organisation, NUS Business School, National University of Singapore, 15 Kent Ridge Drive, Singapore, 119245 Singapore; 2 Department of Management and Organizational Development, D’Amore-McKim School of Business, Northeastern University, 370 Huntington Ave, Boston, MA 02115 USA; 3 Department of Management, ESCP Business School – Berlin Campus, Heubnerweg 8-10, 14059 Berlin, Germany; 4 Rotterdam School of Management, Erasmus University Rotterdam, Burgemeester Oudlaan 50, 3062 PA Rotterdam, The Netherlands; 5 Department of Developmental, Personality and Social Psychology, Ghent University, Henri Dunantlaan 2, 9000 Ghent, Belgium

**Keywords:** Psychology, Human behaviour

## Abstract

The use of chatbots is becoming widespread as they offer significant economic opportunities. At the same time, however, customers seem to prefer interacting with human operators when making inquiries and as a result are not as cooperative with chatbots when their use is known. This specific situation creates an incentive for organizations to use chatbots without disclosing this to customers. Will this deceptive practice harm the reputation of the organization, and the employees who work for them? Across four experimental studies, we demonstrate that prospective customers, who interact with an organization using chatbots, perceive the organization to be less ethical if the organization does not disclose the information about the chatbot to their customers (Study 1). Moreover, employees that work for an organization which requires them to facilitate the deceptive use of a chatbot exhibit greater turnover intentions (Study 2) and receive worse job opportunities from recruiters in both a hypothetical experimental setting (Study 3) and from professional job recruiters in the field (Study 4). These results highlight that using chatbots deceptively has far reaching negative effects, which begin with the organization and ultimately impact their customers and the employees that work for them.

## Introduction

When a Google engineer proclaimed that LaMDA—Google’s chatbot technology^1^—had achieved the status of a “sentient” being^2^, it sparked immense debate worldwide among experts and attracted considerable attention to chatbots and their capabilities. Although the claim itself has been regarded as controversial^3^, one implication is very clear. Chatbot technology today has reached a remarkable level of human-like fluency, tone, and conversational prowess. A chatbot is a computer program, using Natural Language Processing (NLP), that is designed to converse directly with human-end users either via text chats or voice commands^4^. In parallel with these remarkable developments in chatbot technology is the accelerating adoption of chatbots in organizations to handle customer service inquiries^5,6^. Indeed, more than two thirds of executives in service-oriented organizations report that they are proactively looking to adopt chatbots to provide customer-related services^7^. Since chatbots provide immense cost cutting opportunities, allow organizations to provide round-the-clock service provision, and have reached a remarkable level of human likeness, it is easy to understand why many organizations are adopting this emerging practice.

Although chatbots can significantly increase the (economic) efficiency of organizations, customers in fact report negative experiences when engaging with chatbots rather than with humans^8^. In addition, these reactions worsen when the human likeness of chatbots is very high, a phenomenon referred to as the uncanny valley^9^. After all, humans deeply value human interaction^10^ and this is especially true when their own preferences and financial decisions are implicated, as is the case for service provision^11^. However, these negative effects do not emerge if customers are deceived into believing they are interacting with another human and not a chatbot^12–14^. Organizations today therefore face a serious dilemma regarding how to present and usechatbots when handling customer service inquiries. Specifically, they observe that chatbots can—from an economic point of view—improve the efficiency of the organization by reducing costs and provide round-the-clock customer service. At the same time, however, they also realize that if customers know that the service agent is a chatbot and not a human, customers will react negatively. Companies today are therefore confronted with a choice of whether to stay silent towards customers about their use of chatbots and secretly reap the economic benefits or be open about it and possibly suffer negative consequences.

**Figure 1:**
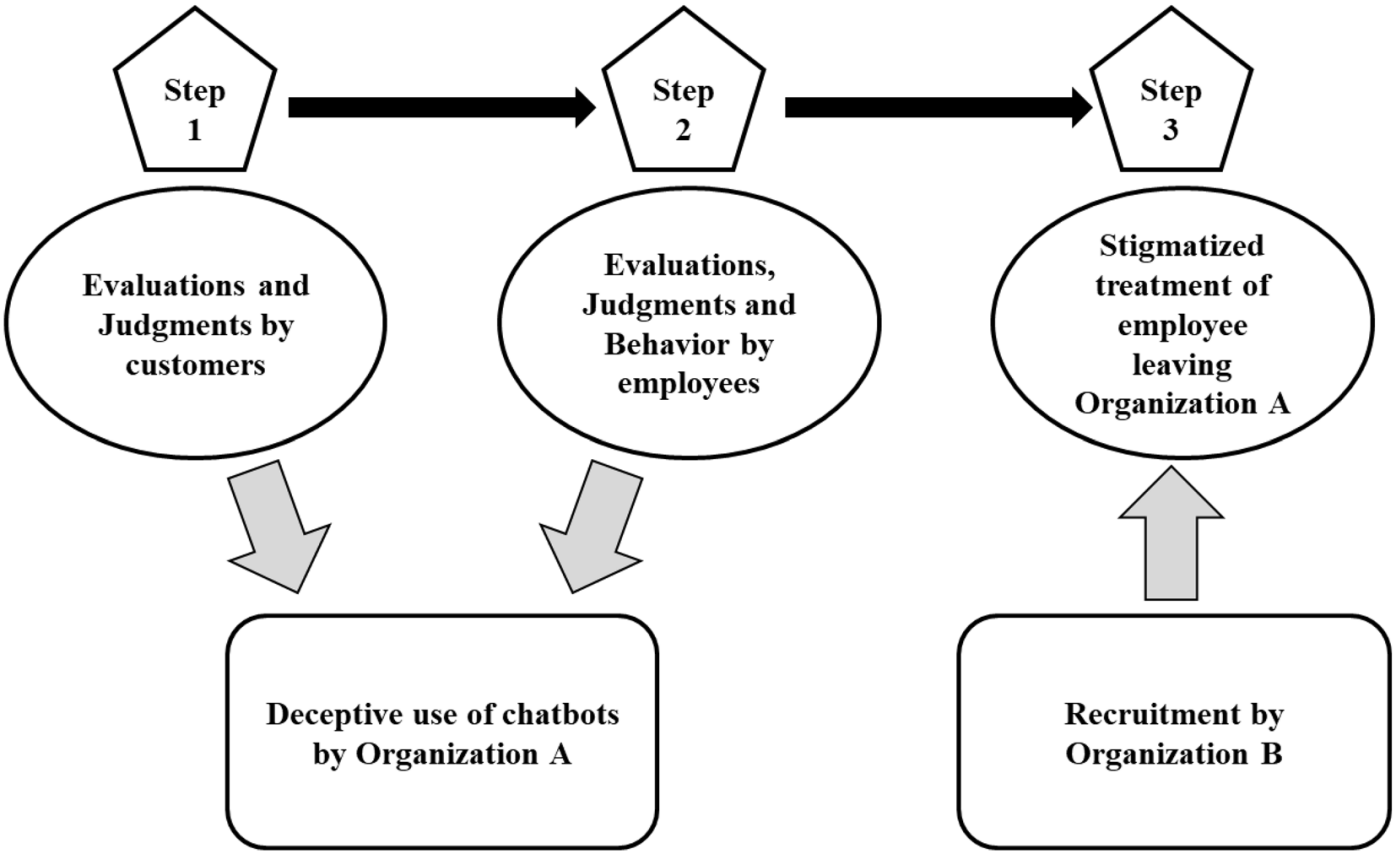
Three-step framework for evaluating the reputational and ethical consequences of deceptive chatbot use.

In our research, we investigate the potential range of negative consequences that may ensue when an organization opts to use chatbots to handle customer service inquiries, but intentionally chooses not to convey this information to its customers. To do so, we adopt a three-step framework (see Fig. 1). Our three-step framework investigates both proximal and distal consequences of deceptive chatbot use. As a first step, we measure the effect of deceptive chatbot use on the evaluations and judgments of prospective customers. In doing so, we capture the immediate impact deceptive chatbot use has on the end-user and reveal its proximal consequences. Step 2 broadens our scope and reveals the distal consequences of deceptive chatbot use by investigating the impact this practice has on the evaluations, judgments and behavior of the employees working for such organizations. Finally, step 3 broadens our scope of distal consequences even further by testing how employees of this deceptive organization will be treated and evaluated by recruiters if they were to leave and seek out a new job position elsewhere.

## Theory and hypotheses development

Despite the fact that chatbots today can demonstrate remarkable human-like qualities^1,15^, their effectiveness is significantly reduced when the human end-user becomes aware that they are interacting with a machine and not a human^12,13^. Why would this be the case? First, people penalize algorithms, a form of machine intelligence, more than humans when errors are committed, which in turn deteriorates the extent to which a person is willing to rely on the algorithm over time^16,17^. As chatbots are bound to display service failures at one point or another^18^, it follows that the human end-users will penalize the chatbots more severely when compared with a human operator, reducing the customers’ willingness to cooperate with it. In further support of our line of reasoning, other research has also shown that humans have a tendency in general to trust other humans more than they trust machines^12,19^, and that the more trust customers are willing to give, the more effective the communication that takes place will be^20^. Therefore, when it comes to service provision, humans prefer human agents over machine-based agents, and this is particularly true when they inevitably err^17^.

All of this suggests that when the technology is at a level where chatbots are indistinguishable from humans, organizations will face a strong temptation to deceive their customers. As the example of LaMDA^1^ illustrates, this stage of technological sophistication is a reality. As such, organizations are now in a position to hide from their customers that chatbots are handling their customer inquiries. Such an approach is obviously problematic because deceiving customers, if found out, is likely to bounce back and result in customers evaluating the organization in negative ways. Of course, any organization will prefer to foster positive customer perceptions, but the fact that automating service delivery has the enormous potential to bring significant financial gains may motivate acts of deception, nonetheless. As such, deceptive tactics have the potential to harm the reputation of the organization, possibly beyond repair. It is therefore crucial for organizations to understand how customers will react when the deceptive use of chatbots is revealed. In this research, we capture the reputation of an employee and organization through perceptions of trust and ethics, as trustworthiness and perceived ethicality are critical inputs when judgments about a person/organization’s reputation are formed^21^. In addition, we define reputational consequences as behavioral outcomes that result from the negative reputation (unethical and untrustworthy) of an employee/organization. We focus explicitly on employees’ desire to leave the organization (Study 2) and recruiters’ hiring decisions (Studies 3 and 4) as reputational consequences of deceptive chatbot use.

### Deceptive chatbot use and customers’ reactions

Understanding how an organization’s potential customer base reacts to deceptive chatbot use is critically important for the organization’s reputation^21^. Additionally, choosing not to disclose the use of a chatbot is an act of deception and although there is a myriad of ways in which trust can be violated, deception is a particularly potent way to do this^22–25^. Of course, organizations that deceive will wish that their transgressions are not known publicly, but these acts—for example due to whistleblowing^26^—can become public knowledge. When these deceptive actions become public knowledge, this has been shown to negatively affect relationships^27,28^, trigger retaliatory responses^29^, and produce negative emotions^30^. And, although research shows that reparations can be made when trust has been violated^31^, a violation of trust that involves intentional deception has been shown to produce an enduring harm to trust which may well be beyond repair^24^.

In addition, there is also an ethical component to deceptive chatbot use as deception breaks ethical norms and codes of conduct, reveals information about the ill-intentions of the deceiver, which undermines respect and liking between the two parties, and ultimately produces a reciprocal relationship of dishonesty^27,28,32^. When an organization utilizes chatbot technology secretly, they are willfully misrepresenting information with the intent to encourage customers to make incorrect conclusions about who they are interacting with^33^. As such, this action carries significant ethical weight^34^ and communicates that one does not want to be truthful. In turn, this violates the principle of transparency, which involves the concealment of relevant information^35^. Being truthful and transparent are thus considered important ethical dimensions^36^. Two of the most important means by which deception leads to a damaged organizational reputation is via perceptions that the organization is untrustworthy and unethical. Indeed, perceptions of trust and ethics contribute greatly to the notion and extent of an organization’s reputation^37^. Based on the above insights, we propose the following hypotheses for the customer perspective (Step 1 in Fig. 1):

#### H1a

Organizations that use chatbots deceptively (vs. transparently) will be trusted less by prospective customers.

#### H1b

Organizations that use chatbots deceptively (vs. transparently) will be perceived to be less ethical by prospective customers.

### Deceptive chatbot use as an unethical request

As part of our second step, we extend our scope by examining how employees working for an organization that requires them to deceive customers about the use of chatbots will affect the way they perceive the organization and their desire to continue working for them. Employees are needed by organizations to carry out organizational practices^38^ and using chatbots in deceptive ways is no different. When not informing customers about the robotic nature of the service provider (and letting the idea that a human is servicing them remain alive), employees will feel that they are breaking their client’s trust and potentially damaging the relationship they share^39^. This situation is evident when considering that many organizations adopt what is known as a chatbot-human hybrid model^40^. In such systems, human operators monitor customer interactions with chatbots and intervene only when the chatbot encounters problems. In cases such as these, customers are unaware of the chatbot nature of their customer service agent and the human employee is needed to deceptively smooth over any issues that arise, maintaining the illusion of purely human customer service.

Employees may agree or not with this organizational practice, but usually they have little voice in this process, meaning that employees will likely feel pressured into committing such unethical acts^41^. In fact, according to a National US Survey of more than 14,500 employees, nearly one in four people (23%), feel pressure to compromise their own ethical standards to fulfil their job roles^42^. Therefore, before examining the effects associated with employees receiving directives to commit unethical acts, it’s important to consider why their compliance with such directives is likely. First, employees are likely to follow through on unethical directives due to the existing power imbalance that exists between employees and their employer^43^. This is because saying “no” has been found to be more uncomfortable for employees than following through with committing the requested unethical deeds^44^. Understandably, there is good reason to refrain from objecting to these directives because not adhering to organizational (unethical) practices can lead to fewer rewards and career opportunities further down the line^45^. Such reasons for adhering to unethical organizational practices will influence the perceptions of employees towards the organization when it comes down to complying with the organizational request to not tell customers a chatbot is being used.

When employees experience pressure from an organizational authority to behave in ethically questionable ways, as is the case with perpetuating deceptive chatbot practices, this is typically followed by negative attitudes and behaviors towards the enacting authority^46^. For employees in this situation, being required to engage in ethically questionable behavior ultimately leads to a loss of trust as it reflects a lack of ethical standards and principles^47^. Indeed, when the organization is perceived by employees to have an unethical work climate, this not only harms perceptions that the organization is an ethical one, but also damages trust between the employees and those who manage them^48^.

We also argue that when an organization requires their employees to comply with these deceptive chatbot practices, employees will perceive this to be an unethical request. An unethical request is a directive issued from an organizational authority to an employee to commit actions that violate social and moral norms^49^. Since being required to conceal the true identity of a chatbot agent involves deceiving customers, thereby violating the ethical principle of transparency and sharing relevant information^35^, employees will indeed be likely to consider this request from the organization to be an unethical one. This perception can also impact important employee outcomes, such as the intention to leave the organization as expectations to engage in immoral conduct can elicit feelings of anger among employees^50^ and negatively impact intrinsic job motivation^51^. Relatedly, when employees believe they are working for an organization that requires them to commit unethical deeds, they no longer perceive the organization to be an effective one^52^ and do not support the leaders that represent them^53^. These findings are consistent with prior research that has found that unethical work climates and the presence of unethical leaders result in greater employee turnover^48,54,55^. This brings us to our next set of hypotheses (Step 2 in Fig. 1):

#### H2a

Employees working for an organization which requires them to use chatbots deceptively (vs. transparently) will exhibit less trust towards the organization, will perceive the organization to be less ethical, and will perceive this request to be unethical.

#### H2b

Employees working for an organization which requires them to use chatbots deceptively (vs. transparently) will exhibit greater intentions to leave the organization.

#### H3

The positive effect of employees working for an organization which requires them to use chatbots deceptively (vs. transparently) on their intention to leave the organization will be mediated by greater perceptions that this is an unethical request.

### Employee moral spillover and future job opportunities

So far, we have reasoned that the deceptive use of chatbots will harm the reputation of the organization as both customers and employees will perceive the organization in negative ways (untrustworthy and unethical). We extend this line of reasoning by also arguing that the personal reputation of the employee may suffer when wanting to leave the organization (Step 3 of Fig. 1). Specifically, as we expect employees to be more willing to leave the organization and look for another job when they are required to facilitate deceptive chatbot use, we assume that their affiliation with the organization may hamper their job search. Building on research that unethical behavior committed by an organization is considered diagnostic of the organizational group’s moral compass^56^, it stands to reason that an individual employee will be evaluated more negatively (less trustworthy) when they have worked for a company with this type of reputation. When a higher-ranking organizational member (i.e., someone who is usually perceived as representing the organizational culture) engages in unethical acts, people perceive this behavior to be prototypical, leading them to perceive others in that organization as unethical too^57^. Thus, how unethical an organization is perceived to act has the potential to color how unethical and trustworthy its employees are perceived as well^58^. That is, when a job candidate shares a history with a company known to use deceptive chatbot practices, potential employers will likely trust this candidate less and feel more uncertain about hiring them^59^.

This spillover process therefore affects the job chances for those employees who want to leave the organization because of the unethical requests they were subject to. Indeed, subordinates working under a higher-ranking organizational member that had committed an unethical act were less likely to receive job-hiring recommendations when compared with a candidate who was merely known to work with a lower-level peer that had committed the same unethical act^57^. These spillover effects not only occur between employees at work^60^, but even companions of the stigmatized employee are negatively affected as well—a phenomena referred to as a stigma-by-association effect^61^. We focus on perceptions of trust specifically as the mediating mechanism linking affiliation to a deceptive organization and subsequent hiring decisions for several important reasons. First, trustworthiness signals whether employees will be cooperative, loyal, and committed^62^. Second, job recruiters are significantly influenced by whether they consider a candidate to be trustworthy when making job-hiring decisions, and even go so far as to measure it in their selection process^63,64^. Indeed, “trust is essential for initiating, maintaining, repairing, and elevating social relationships at work”^65^^, p. 248^, rendering it of crucial importance to organizations and the judgments of recruiters. Finally, trust is intimately related to people’s willingness to take risk, such that trust promotes risk taking in relationships^62^. Hiring decisions inherently involve taking risk because recruiter’s cannot be sure how an employee will behave long-term, thus, trust will be a formative judgment in any hiring decision.

In addition to fewer job-hiring recommendations, we reason that this negative spillover effect may also materialize in other ways. Specifically, if employees from a deceptive organization will nevertheless receive a job offer, it may well be that they will be offered a lower salary. A lower salary is often regarded as signaling less respect and lower status conferral in society and organizational settings^66,67^, and individuals that are associated with morally questionable behavior are usually afforded lower status and respect^68^. Therefore, potential job candidates that have a history with working for a deceptive organization will be likely to receive a lower salary if they were to be extended a job offer.

All of this leads us to our final set of predictions (Step 3 in Fig. 1):

#### H4a

Recruiters will exhibit less trust towards a candidate that has previously worked for an organization which uses chatbots deceptively (vs. transparently).

#### H4b

Recruiters will be less likely to extend a job offer to a candidate that has previously worked for an organization which uses chatbots deceptively (vs. transparently).

#### H4c

Recruiters will offer a lower salary to a candidate that has previously worked for an organization which uses chatbots deceptively (vs. transparently).

#### H5

The negative effect of a candidate having previously worked for an organization which uses chatbots deceptively (vs. transparently) on the likelihood that a recruiter will extend a job offer will be mediated by lower levels of trust towards that candidate.

## Overview of studies

We conducted four experimental studies (including one experiment in the field) to test our hypotheses. In Study 1, we tested how customers perceive organizations which use chatbots deceptively versus transparently. Specifically, we measured their trust towards the organization and how ethical they perceived this organization to be. In Study 2, we investigated the evaluations and perceptions of the employees having to work with the organizational practice of being transparent or not about the use of a chatbot towards their customers. Specifically, we measured their perceptions of whether their organization was making an unethical request to them, their perceptions of trust and ethics towards the organization, and their willingness and decision to leave the organization. In Study 3, participants were placed in the role of a hiring Senior Manager and shown the profile of a job candidate having worked for an organization that uses chatbots deceptively (vs. transparently). Participants were then asked to indicate whether they would extend a job offer to this candidate, evaluate the candidate’s trustworthiness, and the salary they would offer this candidate. Study 4 was set out to replicate the findings of Study 3, but at the same time, extend the design and methodology in important ways. Specifically, we conducted an experiment in the field in which we used real professional job recruiters as our study respondents, as such strengthening both the external and internal validity of our findings^68^. Data across all studies were analyzed using SPSS (version 28). As part of a robustness check, we also ran two-stage least squares (2SLS) regression analyses with our experimental manipulations as our instruments and found the same pattern of mediating effects (see Appendix A). Analyses using SPSS are reported in the manuscript.

## Study 1

Study 1 was a between-subjects experimental design. In this study, participants either encountered an organization that was known to use chatbots deceptively (deceptive condition), transparently (transparent condition), or no such information regarding whether the use was deceptive or transparent was provided (control group). Participants provided ratings of how ethical and trustworthy they perceived the organizations to be (using two validated scales).

### Results

A series of one-way ANOVAs and LSD tests were conducted. Following current recommendations, we conducted analyses both with, and without, a series of control variables: age, gender, work hours per week, and years of work experience^69^. The primary results presented below reflect analyses with control variables. The pattern of results do not change when performing analyses without control variables. Means, standard deviations, and statistical test results are included in Table 1. Results indicated that participants in the deceptive condition perceived the organization to be less ethical (*p* < 0.001) and less trustworthy (*p* = 0.001), relative to those assigned to the transparent condition. However, perceptions of trust in the control condition and deceptive condition did not differ (*p* = 0.156), although perceptions of organizational ethicality did differ between the control and deceptive conditions (*p* = 0.023). These results suggest that transparency may be needed for customers to exhibit greater trust in the organization.

**Table 1:** Means, standard deviations, and LSD tests (Study 1).

	Means and standard deviations					
	Control condition	Transparent condition	Deceptive condition	Df	F	Cohen’s f
Trust in the organization	4.41_a_ (0.98)	4.90_b_ (1.11)	4.06_a_ (1.28)	2,147	6.167**	0.27
Organizational perceived ethicality	4.39_a_ (1.17)	5.10_b_ (1.01)	3.83_c_ (1.43)	2,147	12.797***	0.40

Values in brackets refer to standard deviations. Within a row, values with different subscripts are significantly different (*p* < 0.05) and values sharing the same subscripts are not significantly different (*p* > 0.05), based on LSD tests. One-way ANOVA and LSD tests include age, gender, work hours per week, and years of work experience as control variables. * *p* < 0.05, ** *p* < 0.01, *** *p* < 0.001.

Therefore, we do not find full support for H1a, but we do find support for H1b.

## Study 2

Study 2 was a between-subjects experimental design. Participants occupied the role of an employee in a simulated organization and were either expected by their Senior Manager to be deceptive (deceptive condition) or transparent (transparent condition) towards incoming customers about the use of a chatbot to handle their inquiries. A third condition did not provide participants with any information regarding the deceptive or transparent nature of the chatbot use (control group). Participants provided ratings of their unethical request perceptions, how ethical and trustworthy they perceived the simulated organization to be, and their turnover intentions (using both a validated scale and behavioral measure).

### Results

A series of one-way ANOVAs, LSD tests, and logistic regressions were conducted. The primary results presented below reflect analyses with a series of control variables: age, gender, work hours per week, and years of work experience. The pattern of results do not change when performing analyses without control variables. Table 2 reports the findings for trust in organization, organizational perceived ethicality, turnover intentions, and perceived unethical request. Table 3 summarizes the findings of the binary-choice question that pertains to turnover intentions.

**Table 2:** Means, standard deviations, and LSD tests (Study 2).

	Means and standard deviations					
	Control condition	Transparent condition	Deceptive condition	Df	F	Cohen’s f
Unethical request	–	1.74_a_ (1.25)	4.04_b_ (1.61)	1, 112	73.02***	0.79
Turnover intentions	2.67_a_ (1.61)	2.17_a_ (1.33)	3.53_b_ (2.04)	2, 169	9.89***	0.32
Trust in the organization	4.48_a_ (1.17)	4.77_a_ (1.33)	3.69_b_ (1.23)	2, 169	10.99***	0.35
Organizational perceived ethicality	4.61_a_ (1.21)	5.03_a_ (1.43)	3.59_b_ (1.31)	2, 169	17.31***	0.44

Values in brackets refer to standard deviations. Within a row, values with different subscripts are significantly different (*p* < 0.05) and values sharing the same subscripts are not significantly different (*p* > 0.05), based on LSD tests. One-way ANOVA and LSD tests include age, gender, work hours per week, and years of work experience as control variables. * *p* < 0.05, ** *p* < 0.01, *** *p* < 0.001.

**Table 3:** Cross tabulation of behavioral measures (Study 2).

	Frequencies and percentages of those indicating “yes”				
	Control condition	Transparent condition	Deceptive condition	Df, N	X^2^
‘If given the opportunity, I would leave SPARK for another company’	11_b_ (18%)	6_b_ (10%)	31_a_ (53%)	2, 178	31.69***

Values in brackets refer to percentages. Within a row, values with different subscripts are significantly different (*p* < 0.05) and values sharing the same subscripts are not significantly different (*p* > 0.05), based on LSD tests. * *p* < 0.05, ** *p* < 0.01, *** *p* < 0.001.

First, employees considered the organization to be less trustworthy in the deceptive condition than in both the transparent (*p* < 0.001) and control condition (*p* = 0.006). The same pattern of results was also found for perceptions of organizational ethicality when compared with both the transparent (*p* < 0.001) and control group (*p* = 0.002). Furthermore, employees considered the expectation to use chatbots deceptively to be an unethical request, relative to those that were not requested to use chatbots deceptively (*p* < 0.001). These results suggest that for employees the act of deceiving is what drives the negative effects on trust in the organization and ethics perceptions. Participants also exhibited the greatest turnover intentions in the deceptive condition, relative to both the transparent (*p* < 0.001) and control conditions (*p* = 0.006).

With respect to our binary choice measure, results revealed that those in the deceptive condition, relative to the transparent (χ^2^ = 13.23, *p* < 0.001, odds ratio = 10.33) and control conditions (χ^2^ = 3.43, *p* < 0.001, odds ratio = 5.11), were more likely to indicate that they would leave the organization, if given the opportunity (Table 3).

In addition, we also tested whether the perception of an unethical request mediated the effect of deceptive chatbot use on the decision to leave the organization. To test this mediating effect, analyses were conducted using Model 4 in SPSS with PROCESS, a bootstrapping application provided by Hayes^70^ which enables the significance testing of indirect effects via bias corrected confidence intervals. The tested model includes age, gender, work hours per week, and years of work experience as control variables. The pattern of results do not change when performing analyses without control variables. Results are based on 10,000 bootstrapped samples. Deceptive chatbot use was positively associated with unethical request perceptions (B = 2.29, *p* < 0.001), whereas unethical request perceptions was positively associated with the decision to leave the organization (B = 1.00, *p* < 0.001). Next, the confidence interval for this indirect effect excluded zero, indicating that unethical request perceptions partially mediated the effect of deceptive chatbot use on decision to leave the organization (indirect effect = 2.29, 95% bootstrapped CI = [1.49; 4.75]). Finally, the direct effect of deceptive chatbot use on decision to leave was not significant (B = 0.91, *p* = 0.16). Overall, findings in Study 2 provided support for H2a, 2b, and 3.

## Study 3

Study 3 was a between-subjects experimental design. Participants occupied the role of a Senior Manager in a simulated organization that was tasked with evaluating a job candidate that had either previously worked for a company that used chatbots deceptively (deceptive condition) or transparently (transparent condition). Participant managers provided ratings of the degree to which they trust the job candidate, whether they would hire the job candidate, and the salary they would offer the job candidate (using both validated scales and a behavioral measure).

### Results

A series of independent T-tests and logistic regressions were conducted (see Tables 4 and 5).

**Table 4:** Independent samples t-test results (Study 3).

	Means and standard deviations				
	Transparent Organization condition	Deceptive Organization condition	Df	F	Cohen’s d
Trust in candidate	3.82 (0.87)	3.07 (0.91)	1,102	18.10***	0.84
Salary offered	42.21 (37.09)	14.23 (30.40)	1,102	17.79***	0.83

Values in brackets refer to standard deviations. * *p* < 0.05, ** *p* < 0.01, *** *p* < 0.001.

**Table 5:** Cross tabulation of decision to hire candidate (Study 3).

	Frequencies and percentages of those indicating “yes”				
	Transparent organization condition	Deceptive organization condition	Df, N	X^2^	OR
Do you want to hire this candidate	27 (69.2%)	12 (31%)	1, 104	20.01***	0.15

Values in brackets refer to percentages. OR = Odds ratio. * *p* < 0.05, ** *p* < 0.01, *** *p* < 0.001.

First, results show that candidates that previously worked in an organization requiring them to be deceptive (vs. transparent) about their use of chatbots were perceived to be less trustworthy by the middle manager recruiters. To test our mediating hypothesis, analyses were conducted in SPSS (Model 4) with PROCESS and are based on 10,000 bootstrapped samples^70^. The tested model includes age, gender, work hours per week, and years of work experience as control variables. The pattern of results do not change when performing analyses without control variables. We found that candidates that came from organizations that used chatbots deceptively were trusted less (B = 0.77, *p* < 0.001), whereas trust in the candidate was positively associated with offering them a position (B = 1.36, *p* < 0.001). Next, the confidence interval for this indirect effect excluded zero, indicating that trust in the candidate partially mediated the effect of affiliation with an organization that used chatbots deceptively on decision to offer a position (indirect effect = 1.04, 95% bootstrapped CI = [0.52; 2.22]). Finally, the direct effect of affiliation with an organization that used chatbots deceptively on decision to offer a position was significant (B = 1.50, *p* = 0.01). Candidates that came from organizations that used chatbots deceptively were less likely to be offered the position by our middle manager recruiters (χ^2^ = 20.01, *p* < 0.001, odds ratio = 0.15). Therefore, candidates that worked for organizations using chatbots deceptively were perceived as less trustworthy, which in turn reduced their chances of being offered the job position. Finally, an independent samples T-test revealed that our middle manager participants also offered candidates in the deceptive condition a lower salary (M = 14.23, SD = 30.40), relative to those in the transparent condition (M = 42.21, SD = 37.09; *p* < 0.001, Cohen’s d = 0.83). Converting our units into Pounds Sterling (£), this equates to a difference of £1.40.

## Study 4

Study 4 was a between-subjects experiment in the field, utilizing responses from real professional job recruiters. Professional job recruiters were asked to evaluate a job candidate that had either previously worked for a company that used chatbots deceptively (deceptive condition) or transparently (transparent condition). Professional job recruiters, as in Study 3, provided ratings of the degree to which they trust the job candidate, whether they would hire the job candidate, and the salary they would offer the job candidate (using both validated scales and a behavioral measure).

### Results

A series of independent T-tests and a logistic regression were conducted. The results of these tests, including means and standard deviations, are found in Tables 6 and 7.

**Table 6:** Independent samples t-test results (Study 4).

	Means and standard deviations				
	Transparent condition	Deceptive condition	Df	T	Cohen’s d
Trust in candidate	4.62 (.89)	3.39 (.85)	1, 48	5.01***	1.41
Salary offered	6364.00 (1388.91)	5300.00 (1406.83)	1, 48	2.69**	0.76

Values in brackets refer to standard deviations. * *p* < 0.05, ** *p* < 0.01, *** *p* < 0.001.

**Table 7:** Cross tabulation of decision to hire candidate (Study 4).

	Frequencies and percentages of those indicating “yes”				
	Transparent condition	Deceptive condition	Df, N	X^2^	OR
Do you want to hire this candidate	22 (88%)	6 (24%)	1, 50	22.69***	0.27

Values in brackets refer to percentages.

*OR *odds ratio.

Our findings reveal that professional job recruiters significantly penalize job candidates who in the past worked for organizations that used chatbots deceptively. We found that candidates that came from organizations that used chatbots deceptively were trusted less (B = 1.29, *p* < 0.001), whereas trust in the candidate was positively associated with offering them a position (B = 1.06, *p* = 0.05). Next, the confidence interval for this indirect effect excluded zero, indicating that trust in the candidate partially mediated the effect of affiliation with an organization that used chatbots deceptively on decision to offer a position (indirect effect = 1.37, 95% bootstrapped CI = [0.27; 6.32]). Finally, the direct effect of affiliation with an organization that used chatbots deceptively on decision to offer a position was significant (B = 2.24, *p* = 0.01). Candidates that came from organizations that used chatbots deceptively were less likely to be offered the position by our middle manager recruiters (χ^2^ = 22.69, *p* < 0.001, odds ratio = 0.27). To test this mediating effect, analyses were conducted in SPSS with PROCESS^70^. The tested model includes age, gender, and organizational tenure as control variables. The pattern of results do not change when performing analyses without control variables. Results are based on 10,000 bootstrapped samples. Therefore, as in Study 3, candidates that worked for organizations using chatbots deceptively were perceived as less trustworthy, which in turn reduced their chances of being offered the job position. Finally, an independent samples T-test revealed that our professional recruiters offered candidates from a deceptive organization a lower salary (M = 5300.00, SD = 1406.83), relative to those who came from a transparent organization (M = 6363.00, SD = 1388.91; *p* < 0.001, Cohen’s d = 0.76). This equates to a difference of 1,064RMB additional income per month for those who come from organizations that use chatbots in a transparent way.

## General discussion

Across four experimental studies, we found consistent support for our hypotheses. Specifically, we found that organizations that use chatbots deceptively are perceived by prospective customers to be less ethical (Study 1). Interestingly, perceptions of trustworthiness were only significantly different from the control group when the organization was transparent about its chatbot use (as opposed to deceptive), showing the importance of transparency for customers. In addition to this, the nature of chatbot use in organizations influences employees too. Employees that were required to use chatbots deceptively by their organization perceived this request to be an unethical one. They also perceived their organization to be less trustworthy and ethical, and exhibited greater turnover intentions (Study 2). In Study 2, we also found that the negative effect of deceptive chatbot use on the decision to leave the organization was partially explained by greater perceptions that an unethical request was present. Although employees may show a greater desire to leave such an unethical environment behind, we also discovered that their prior employment negatively influenced their future job prospects. Specifically, we found that both temporary job recruiters in an experimental setting (Study 3) and professional job recruiters in the field (Study 4) were less likely to extend a job offer to a candidate from a deceptive organization and this was explained by a perceived lack of trustworthiness. Furthermore, recruiters also offered them a lower salary. Taken together, the findings of our studies provided strong support for our hypotheses.

### Theoretical contributions

First, our findings indicate that there are varying concerns regarding transparency and deception among customers and employees. In Study 1, both the control group and the deceptive condition displayed similar levels of trust towards the organization, while the transparent condition exhibited the highest level of trust. Conversely, Study 2 revealed that employees in the control and transparent conditions held similar perceptions of trust and ethicality towards their organization, whereas those in the deceptive condition demonstrated significantly lower levels of trust and ethics. These results suggest that customers are strongly influenced by the principle of transparency and the provision of relevant information by the organization^35^. This aligns with previous research that highlights a positive relationship between organizational information flow and customer trust^71^. On the other hand, employees' trust and ethics perceptions appear to be negatively affected when customers are deceived, potentially due to their involvement in the deception process^72^. As employees are positioned with knowledge of the business processes and are often required to participate in customer deception, their perspectives differ from those of customers. Consequently, our contribution to theory lies in providing a nuanced understanding of the factors driving negative perceptions among customers and employees in relation to the use of organizational chatbots.

With respect to employees that facilitate the deceptive use of chatbots, we have identified two processes by which this practice negatively impacts them. When employees are required by their organization to use chatbots without disclosing their use to customers, employees perceive this to be an unethical request. Research on unethical requests is still in its infancy^49,73^ and we extend this literature by showing that unethical requests come accompanied with greater employee turnover intentions and lower trust in the organization. Whereas prior research has focused on the general effects of ethical leadership and climate on turnover intentions^54^, we find the first evidence—to our knowledge—of the effect of unethical requests on employee’s decision to leave the organization.

The second process by which employees are negatively impacted concerns their future employment prospects. This is of great theoretical importance because it, first, highlights the far-reaching negative effects of organizations using chatbots deceptively. The use of chatbots in a deceptive manner has negative consequences for all stakeholders at each level: the organization, the employees, and the customers. Second, existing research on moral spillover effects have so far only tested these effects between individuals^57^, whereas we demonstrate that moral spillover can occur between organizations and individual employees. Our work also departs from research on organization-level effects and outcomes of stigma-by-association^74^ and instead focuses on how stigmatized organizations negatively impact their employees, even if such employees want to leave.

### Practical implications

Our results are highly relevant and important for our current organizational climate, as examples of violations of trust and ethics in the deployment of artificial intelligence by organizations are becoming increasingly pervasive^75^. To this end, we believe there are two key practical insights that can be obtained from our work. First, although organizations are highly incentivized to use chatbots for service provision, they should ensure that they do not conceal willingly this information from the end-users who interact with them. Although people have been found to cooperate with and are persuaded by a chatbot when they are led to believe it is actually a human^12,13^, our research indicates that there are great reputational costs that come with concealing their true identity. We therefore strongly recommend that organizations are forthright about their chatbot use and this can simply be done by having the chatbot announce the true nature of its identity at the beginning of every service inquiry interaction. By default, customers will expect a human operator^76^, although it is more commonly accepted now that intelligent technologies can be used when the customer inquiry is routine and repetitive nature, as is the case for checking bank balances or making payments^77^. Therefore, to avoid deceiving customers, organizations will need to be explicit about their use of chatbots, and preferably explain the use of this technology in light of the tasks it performs, if they wish to avoid unwanted and negative reputational consequences.

Second, our research shows that using chatbots deceptively also negatively affects employees in two ways. Although leaders may be able to coerce their employees into engaging in unethical behavior that is good for the firm^78^, they should not abuse this influence as it may result in high employee turnover. Our work therefore provides further practical insight into why organizations should not encourage their employees to engage in unethical behavior, even if doing so is supposedly good for the organization. In addition, jobseekers should bear in mind that the wrongdoing of an organization can harm their own personal reputation and research in this domain suggests that there is little that can be done to restore the personal losses in reputation^79^. To counter this, job seekers should focus on finding organizations that embody values which align well with their own, creating good person-organization fit^80^. Job seekers should achieve this by being very critical when selecting organizations to work for and researching available information about the organizations practices and work culture.

### Limitations and directions for future research

Of course, this work is not without limitations which highlights several fruitful avenues for future research. First, while our research documents a moral spillover effect, where the reputation of leaving employees is negatively affected by the deceptive actions of the organization they worked for, we did not explore boundary conditions of this effect. For example, leaving employees may choose to signal their ethical values when interviewing for new jobs or even go as far to condemn the actions of their previous employer^81^. Such efforts may have a successful cleansing effect on their reputation. It is also likely the case that whistleblowers who decide to speak out against the unethical conduct of their organization won’t be negatively affected by moral spillover effects^82^. We therefore encourage future research to examine the role employees can play in proactively protecting their own reputations.

Moreover, if the quality of the interaction between customers and chatbots can be significantly improved, organizations will have less of an incentive to conceal their use in customer service provision. For example, the process of anthropomorphism (while maintaining transparency about the chatbot nature of the service provider) could be useful in improving these interactions as people tend to cooperate better with robots that appear to possess human-like qualities^83^. Indeed, robots that have been imbued with a human-like appearance have been shown to promote deeper emotional connections^84^ and promote trust^85^, although excessive human likeness can produce negative reactions^10^. In addition, customer reactions to transparent use of chatbots may vary according to the customer’s technological readiness, technology acceptance, and personal experience with chatbots^86,87^. Future research in this domain therefore needs to disentangle these effects so that scholars can identify the optimal conditions for chatbot-human interaction.

Finally, the present research has methodological limitations worth discussing. The experimental studies conducted do not involve real interactions with a chatbot. It would therefore be worthwhile to examine whether direct experience with deceptive chatbots actually exacerbates the negative effects on trust and ethics further. For example, in Study 1, participants occupy a somewhat third-person perspective where they evaluate an organization based on the information provided. If, however, we collected responses from participants after experiencing being deceived by a company chatbot themselves, their negative reactions may be worse as the actual experience of deception could elicit a stronger emotional response than hypothetical deception^88^. In addition, it is not clear whether our findings regarding the perceptions and behavior of employees and recruiters would alter if examined in an organizational context. For instance, recruiters would typically receive contextualized information about candidates and have the opportunity to meet them in person during the interview process. In Studies 3 and 4, however, the opportunity to obtain this type of information was not possible. Thus, we suggest that future studies investigate the consequences of deceptive chatbot use in non-experimental settings as well.

## Methods

All research studies received ethical approval from the Institutional Review Board (IRB) at the University of Cambridge Human Psychology Research Ethics Committee (Ethical Approval Code: 17/011), and all methods of the reported studies were performed in accordance with the ethical guidelines and regulations of this committee (https://www.bio.cam.ac.uk/system/files/documents/handbook.pdf).

### Study 1

#### Sample and design

To determine our effect size estimate, prior literature on the effects of deception on trustworthiness perceptions was consulted^24,25^, leading us to conservatively account for a medium effect size (Cohen’s f = 0.3). An a priori power analysis suggests that approximately 144 total observations are required to achieve 90% power at an α of 0.05^89,90^. However, to account for participants that fail to pay sufficient attention, we exceeded this target in anticipation of this. A total of 180 adult participants were recruited via Prolific Academic in exchange for GBP 0.70 (ProA; http://www.prolific.ac). The estimated study duration was 5 min. ProA is an online platform explicitly designed for online participant recruitment by the scientific community^91^ and has been empirically demonstrated to provide higher quality data than alternative online platforms^92^. One participant did not complete the experiment and a further 24 participants failed to correctly answer an instrumental attention check and were removed from subsequent analyses (see procedure below). Utilizing attention checks is an important method for enhancing data quality^93^, particularly when utilizing online platforms such as Prolific. Thus, we obtained a final sample of 155 participants. Of the 155 participants, on average, they were 32.94 years old (SD = 8.89), had 11.35 years work experience (SD = 9.31), worked 39.78 h per week (SD = 7.32), 60 were female and 95 were male. Participants were randomly allocated to one of three condition groups. In the first condition, participants were introduced to an organization that uses an audio-based chatbot to respond to customer queries and discloses this information to their customers (transparent condition). The second condition was the same with the exception that the organization does not inform their customers that they are talking to a chatbot (deceptive condition). The third condition served as our control group where participants did not receive any additional information regarding the information customers were provided about the chatbot.

#### Procedure

Participants were told that they would be introduced to an organization that we, the experimenters, had ostensibly recently worked with and the name would be kept confidential for legal reasons. In all conditions, participants were informed that the organization had implemented an audio-based, algorithmic chatbot that is so sophisticated that it is indistinguishable from a real human voice^1,15^, and the intended use of the technology is to serve as a customer service agent for incoming customer inquiries.

In the transparent [deceptive] condition, supplementing this information, participants were also told that:

At the executive level it was decided that customers calling in will be notified first [will not be notified] that they will be speaking with an algorithm that cannot be distinguished from a human voice.

In the control condition, no additional information about whether the organization intended to inform customers about the conversational chatbot was given.

To check if participants sufficiently understood the condition to which they were assigned, we administered an instrumental attention check in the first two conditions asking: “Based on the information you have just read, will the organization notify customers that they will be speaking with an algorithm that cannot be distinguished from a human voice? (Yes/No)”. In total, 24 participants provided answers that were inconsonant with the condition to which they were assigned, leading us to remove their responses from our subsequent data analysis.

#### Measures

For all Likert-scale measures, the scale ranged from 1 (strongly disagree) to 7 (strongly agree).

Trust in the organization. We measured the extent to which participants trusted the organization using a validated 13-item trust scale that was adapted to our experimental context^94^. Sample items include: “I would not need to worry about whether this organization will stick to their word” and “this organization is very concerned about the welfare of their customers” (M = 4.47, SD = 1.15, Cronbach’s α = 0.95).

Organizational ethicality. The perceived ethicality of the organization was measured using a 4-item scale^95^. Scale items included “this organization is a socially responsible company” and “this organization respects moral norms” (M = 4.47, SD = 1.30, Cronbach’s α = 0.90).

Finally, the participants provided demographics, and an instrumental attention check was introduced to ensure we only retain high quality responses. All remaining participants responded to this check correctly. Participants were given the option to comment on the experiment, thanked for their participation, and debriefed.

### Study 2

#### Sample and design

An a priori power analyses was conducted and indicated that a total sample size of 176 would successfully detect valid, medium sized effects across our three experimental conditions (Cohen’s f = 0.3; power = 90; α = 0.05). A total of 240 participants were recruited via Prolific to take part in our experiment in exchange for GBP 1.00. The estimated study duration was 7.5 min. To be eligible for our study, participants needed to be full-time employees and have not participated in Study 1. Of this sample, 2 participants did not complete the experiment and 60 were removed from our analyses after failing to provide sufficient attention. Thus, we obtained a final sample of 178 participants. On average, the 178 participants were 35.81 years old (SD = 9.64), had 15.74 years of work experience (SD = 9.75), worked 38.26 h per week (SD = 7.47), 96 were female and 82 were male. As in Study 1, participants were randomly allocated to three conditions (deceptive condition, transparent condition, control group).

#### Procedure

After agreeing to the informed consent, participants logged into our online platform, chose a personal username, and were told that they will be connected to four other participants via the platform. The in-basket task approach was implemented because this approach provides a realistic and externally valid environment for organizational decision-making and behavior to take place, while also retaining the advantages of conducting research in a controlled environment^96,97^. From this perspective, participants’ responses represent real decision behavior, although the setting in which this takes place is simulated. This setting was created by telling participants that they would be working with other team members for a company called SPARK. We explained that, as in most organizations, SPARK consists of different hierarchical layers and that they will have to execute several tasks. To make this clear, a visual representation of the organization’s hierarchy was provided [see Appendix B;^98^]. They learned that one member will hold the position of senior manager, another will hold the position of middle manager, and that three others will hold employee positions. Once participants understood the set-up, they were ostensibly connected to other participants and seemingly allocated to one of the three positions at random. To make this allocation procedure appear realistic, it was stated that a network connection needs to be established first before participants can be allocated to teams. This connection procedure was visually depicted using a series of continuously streaming loading bars with varying wait time allotted to each loading stage (see Appendix C). In reality, all participants were allocated the position of “Employee 1”. To check whether participants believed they were connected to other participants, we asked them, “Are you now connected with other participants (yes/no)?”. Only eight participants out of 178 responded ‘no’ to this question, indicating that our simulated organization achieved the aim of providing a realistic work setting for our participants.

To provide participants with a rich base of information regarding the organization SPARK, we told participants in detail about how SPARK specializes in designing and promoting financial products for consumers and examples of these were provided. Participants then read that they are responsible for handling incoming customer complaints and that to further aid them, SPARK installed an artificially intelligent chatbot to their interface to facilitate customer complaint responses. Importantly, they learned that the AI chatbot was highly sophisticated and indistinguishable from a human operator in how they communicate. We also informed them that the chatbot was widely adopted among Fortune 500 companies for automating customer service provision, further bolstering the realism of our paradigm. Next, we included an instrumental attention check to test whether participants understood the capabilities of the AI chatbot. Thirty-six participants failed to answer this check correctly and were therefore removed from our analyses. After this we introduced our manipulation. Participants read that their middle manager—who was using the username: Robin90—has just sent them a message:

I am Robin 90. I will be your supervisor while you are developing financial products and interacting with customers. As you know, customers will be able to use our customer service cell in case they have some questions. This cell works with AI that provides answers to these questions in a very realistic and human way.

The message ended here for the control condition. In the deceptive [transparent] condition, the message continued.

I do have to tell you the following important message: The senior manager just told me that SPARK has decided that when customers call in you cannot tell them [have to tell them] that they will be talking to a “chatbot”. The senior manager wants the customers to think [does not want to deceive them by raising the perception] that they are talking to and being helped by an actual human customer service agent.

Therefore, please refrain from mentioning [please mention] that the customer service is a chatbot when communicating with clients.

The context for our experimental paradigm was inspired by real world use of a hybrid system of both human employees and chatbots to perform customer service inquiries^40^. Our simulated organizational paradigm was therefore modelled after this real work dynamic. In both the deceptive and transparent conditions (but not the control group), we asked participants to indicate whether or not senior management told them to inform customers that they will be interacting with a chatbot. Three participants failed this comprehension check and were thus removed from further analyses. In these two conditions, we also asked a few questions regarding their senior manager’s decision to disclose (vs. not disclose) this information to their customers.

#### Measures

For all Likert-scale measures, the scale ranged from 1 (strongly disagree) to 7 (strongly agree).

Unethical requests. We first measured the extent to which subordinates felt that what their supervisor expected from them was an unethical request. We did so by collecting responses for four items from an unethical requests scale developed by Desai and Kouchaki^49^. The items were: my supervisor “asked me to do something that is morally inappropriate”, “asked me to do something that makes me feel dirty afterwards”, “asked me to do something that involves lying to others”, and “makes me treat the customers disrespectfully” (M = 2.87, SD = 1.84, Cronbach’s α = 0.92).

From here, all further measures were collected across all conditions.

Trust in organization. The same scale from Study 1 was adapted to measure trust in the organization (M = 4.32, SD = 1.32, Cronbach’s α = 0.96).

Organizational ethicality. The extent to which participants perceived the organization to be ethical was measured using the same scale from Study 1 (M = 4.43, SD = 1.44, Cronbach’s α = 0.93).

Turnover intentions. We measured turnover intentions via responses to two separate questions. First, using a single item extracted from a validated turnover intentions scale^99^—we asked participants to indicate on a 7-point Likert scale: “Are you thinking about quitting your work for SPARK?” (M = 2.79, SD = 1.76). Next, to measure a behavioral response for participant’s decision to leave, we asked whether their response to the following statement would be yes or no—“If given the opportunity, I would leave SPARK for another company”.

Next, before the supposed group tasks would have commenced, the study terminated prematurely due to a seemingly unexpected technical error. Finally, the participants provided demographics, and an instrumental attention check was introduced to ensure we only retain high quality responses. Twenty-one participants failed to answer this check and so their responses were not retained in our analysis. Participants were given the option to comment on the experiment, thanked for their participation, and debriefed.

### Study 3

#### Sample and design

As in the previous studies, we recruited participants on Prolific and participants needed to be full-time employees and have not participated in Study 1 and 2 to be eligible for this study. An a priori power analyses was conducted and indicated that a total sample size of 98 would successfully detect valid, medium sized effects across our two experimental conditions (Cohen’s d = 0.6; power = 90; α = 0.05). However, due to our instrumental attention check exclusion criteria, a total of 161 participants were needed to meet our sample size objective. Thus, a total of 161 participants were recruited via Prolific in exchange for GBP 1.00. The estimated study duration was 7.5 min. However, 57 responses were removed from our analysis after considering an instrumental attention check that is consistent with Study 1 and 2. Thus, we obtained a final sample of 104 participants. On average, the 104 participants were 37.52 years old (SD = 10.07), had 17.91 years of work experience (SD = 10.09), worked 37.68 h per week (SD = 5.86), 56 participants were female and 48 were male. Participants were randomly allocated to two conditions. In contrast to Study 2, participants were placed in the role of a recruiter and had to assess profiles of candidates who had worked for a company that either used chatbots deceptively (deceptive organization condition) or choose to disclose this information to their clients (transparent organization condition).

#### Procedure

We used the same procedure and in-basket task approach as in Study 2. However, instead of being allocated to the employee position, all participants learned that they would hold the middle manager’s position. As before, by presenting a series of loading pages that simulated the experience of waiting for participants to be connected, we gave participant’s the impression that they were working in a real group task and that they were randomly assigned the position of middle manager. Only six of the total 104 participants responded ‘no’ to whether they were really connected to other participants, further evidencing the realism of our paradigm. Again, we provided participants with a rich base of information regarding the organization SPARK and its business operation. Next, they learned that they had one employee position that was vacant. Importantly, participants were told that they had to decide between two other participants that were connected via our simulated organization platform. Once participants understood the task, they received one of two profiles. In the deceptive (vs. transparent) condition, the candidate’s profile was described in the following way:

This person was a participant in another study that took place not too long ago.

In that study, he/she worked in an organization that served customers using a chatbot that worked with an algorithm that was so sophisticated that people could not distinguish between this algorithm and an actual human service agent in terms of content, phrasing and communication. The norm in this organization was that they never [always] told customers that they were actually talking to an algorithm. In this organization, employees thus deceived [did not deceive] their customers by making them believe they were communicating to a human agent.

To ensure we only retained participants that read the profile carefully, we asked them to indicate whether or not the candidate’s organization disclosed the fact that they were using chatbots. A total of 42 participants failed this quality check by providing responses that were inconsonant with their assigned condition and were thus removed from further analyses.

#### Measures

For all Likert-scale measures, the scale ranged from 1 (strongly disagree) to 7 (strongly agree).

Trust. We subsequently measured the extent to which the participant recruiters trusted the candidate using an adapted scientifically valid trust scale^94^. Sample items from the resulting five-item scale include: “I would not let this candidate have any influence over issues that are important to me” and “If someone questioned this candidate’s motives, I would give this candidate the benefit of the doubt” (M = 3.38, SD = 0.97, Cronbach’s α = 0.76).

Hiring decision. Next, we asked, “Do you want to recruit this individual for the employee position in your team? (yes/no)”.

Salary offer. In addition to this, we asked participants the salary they would like to offer the candidate using a scale from 1 to 100 units (whereby 1 unit = £0.05 and 100 units = £5.00). The pay scale was administered in this way so that the salary would be commensurate with the level of payment participants were receiving for their own participation.

Once participants provided their responses regarding the candidate they evaluated, what appeared to be a technical error prevented them from receiving the profile of the second participant candidate. Finally, the participants provided demographics and an instrumental attention check was introduced to ensure we retain only high-quality responses. Fifteen participants failed to respond to this check adequately and were therefore removed from our analyses. Participants were then given the option to comment on the experiment, thanked for their participation, and debriefed.

### Study 4

#### Sample and design

We used the effect size of the main effects on trustworthiness and salary offered from Study 3 (Cohen’s d = 0.83 and 0.84, respectively) to determine the sample size needed. An a priori power analysis suggests that approximately 50 total observations are required to achieve 90% power at an α of 0.05. A post-hoc power analysis based on the effect size of our experimental manipulation on trustworthiness (Cohen’s d = 0.86), salary offered (Cohen’s d = 0.76), and decision to hire the candidate (Odds Ratio = 0.27), yielded a power of 0.91, 0.84, and 0.95, respectively, making this sample sufficient for hypothesis testing. After receiving help from five Senior Human Resources Partners, a total sample of 50 professional HR recruiters were recruited using a snowball sampling method, all of whom completed the survey voluntarily without monetary compensation. Thus, we obtained a total final of 50 recruiters. The estimated study duration was 6 min. These HR recruiters come from twenty-one major cities in various parts of the Peoples Republic of China (e.g., Beijing, Guangzhou, Shanghai, etc.). This study was first developed in English and translated into Chinese using back-translation procedures^100^. Our professional recruiter sample were, on average, 35.42 years (SD = 6.05), reported a work tenure of 12.06 years (SD = 5.96), 35 were female and 15 were male. The recruiters were randomly assigned to one of two conditions (deceptive organization condition vs. transparent organization condition), as in Study 3.

#### Procedure

After agreeing to the informed consent, participants were asked to evaluate the upcoming study context from the perspective of their position as a professional recruiter. To make this explicit, we told our recruiter participants, “we would like you to act as you do in daily work life when you are involved in the recruitment of potential employees” and “we want to ask you to act in line with your professional obligations as a recruiter when being asked for your opinions and to make decisions”. The objective of making this explicit was to ensure that our responses reflect as closely as possible real recruiter behavior that would occur in real organizational settings. Importantly, we asked participants to exit the study if job recruitment is not a part of their daily job routine. This ensured the validity of our data.

All participants were then asked to indicate their hiring decision about a potential recruit for a service team. The professional recruiters read that the potential recruit worked in an organization that provided a variety of services to customers using a chatbot.

Then, we introduced our experimental manipulation. In the deceptive [transparent] condition, recruiters read that the norm in the organization the job candidate worked for was to never tell [always tell] customers that they are talking to an algorithm. In this organization, employees are known [never known] to be deceiving towards their customers by making them believe they are communicating to a human agent. The materials for this manipulation match those used in Study 3.

#### Measures

For all Likert-scale measures, the scale ranged from 1 (strongly disagree) to 7 (strongly agree). As in Study 3, we then measured from the recruiters the extent to which they considered the candidate trustworthy (M = 4.01, SD = 1.06, Cronbach’s α = 0.83), whether they would offer them the job, and the salary they would offer this candidate. As our sample includes professional job recruiters, we amended the wording of our salary measure so that it was more ecologically valid. Specifically, we asked them, “How much monthly salary would you propose to give to this candidate? Assuming the average salary in this field is 6000 RMB per month?”. This amount was determined after consulting with several senior HR managers in the local region.

Once these measures were collected, participants provided their demographic information, and an instrumental attention check was introduced to ensure we only retain high quality responses, in which all participants successfully passed. Finally, participants were given the option to comment on the experiment, thanked for their participation, and debriefed.

### Ethical approval

All studies research received ethical approval from the Institutional Review Board (IRB) at the University of Cambridge Human Psychology Research Ethics Committee (Ethical Approval Code: 17/011), and all methods of the reported studies were performed in accordance with the ethical guidelines and regulations of this committee (https://www.bio.cam.ac.uk/system/files/documents/handbook.pdf).

### Supplementary Information

Supplementary Information.

## Data Availability

The data that are reported in the present manuscript—including a supplement document containing all questionnaire items and appendices—are made publicly available and can be openly accessed through Open Science Framework: https://osf.io/dce75/?view_only=4864904585044991b842263547553c2e. The data analysis scripts can also be requested from the first author: j.mcguire@u.nus.edu.
