# Supplementary material for: The reputational and ethical consequences of deceptive chatbot use
Source: Sci Rep. 2023 Sep 27;13:16246. doi: 10.1038/s41598-023-41692-3 (PMC10533525; doi:10.1038/s41598-023-41692-3)
Supplement: Supplementary file 1 — Supplementary Information. [file 41598_2023_41692_MOESM1_ESM.docx]

**SUPPLEMENT**

**Questionnaire Items**

**Trust in the organization (Study 1 and 2)**

Please rate the extent to which you agree with the following statements: (1 = strongly disagree; 7 = strongly agree):

- I feel - that SPARK is very capable of helping customers in their calls.
- I feel - that SPARK has much knowledge about how to effectively help customers in their calls.
- I feel - very confident about the skills of SPARK in the service they provide.
- I feel - that SPARK is well qualified at giving their customers help.
- I feel - that SPARK is very concerned about the welfare of their customers.
- I feel - that customer needs are very important to the management of SPARK.
- I feel - that SPARK will go out of their way to help customers.
- I feel - that SPARK would not knowingly do anything to hurt their customers.
- I feel - that SPARK has a strong sense of justice.
- I feel - that I would not need to worry about whether SPARK will stick to their word.
- I feel - that SPARK tries hard to be fair in their dealings with their customers.
- I feel - that sound principles seem to guide the decisions taken by SPARK.
- I feel - that I like SPARK's values.

**Organizational ethicality (Study 1 and 2)**

Please rate the extent to which you agree with the following statements: (1 = strongly disagree; 7 = strongly agree):

- I feel that: - this organization respects moral norms.
- I feel that: - this organization adheres to the law.
- I feel that: - this organization is a socially responsible company.
- I feel that: - this organization is a good company.

**Unethical requests (Study 2)**

Please rate the extent to which you agree with the following statements: (1 = strongly disagree; 7 = strongly agree):

- The supervisor asks me to do something that is morally inappropriate.
- The supervisor asks me to do something that makes me feel dirty afterwards.
- The supervisor asks me to do something that involves lying to others.
- The supervisor makes me treat the customers disrespectfully.

**Trust in candidate (Study 3 and 4)**

Please rate the extent to which you agree with the following statements: (1 = strongly disagree; 7 = strongly agree):

- [R] I would not let have this candidate have any influence over issues that are important to me.
- I would be willing to let this candidate have complete control over my future in this company.
- [R] I would wish I had a good way to keep an eye on this candidate.
- I would be comfortable giving this candidate a task or problem which was critical to me, even if I could not monitor his/her actions.
- If someone questioned this candidate's motives, I would give this candidate the benefit of the doubt.

**APPENDIX A**

We used the ‘ivregress’, ‘ivprobit’, ‘ivreg2’, and ‘gsem’-commands in STATA to run two-stage least squares (2SLS) regression analyses with our experimental manipulations as our instruments. Below, we report the findings from these analyses for each study with mediation-specific hypotheses. Analyses were conducted with a series of control variables (Studies 2 and 3: age, gender, work hours per week, and years of work experience; Study 4: age, gender, and organizational tenure).

**Study 2**

We first ran the Hausman test to assess how strong the potential omitted variable bias is. This test was not significant for turnover intentions (χ^2^(1) = 0.065, p = .798) or the binary choice to quit (χ^2^(1) = 1.013, p = .314). The non-significance suggests that the likelihood of an endogeneity bias is low [2]. Nevertheless, we still report below our findings where we instrumented unethical requests on our manipulations. There was a significant effect of the instrumented ethical request measure on intention to leave the organization (*b = 0.61, SE = 0.12, p <.001)* and the decision to quit the organization (*b = 0.72, SE = 0.12, p <.001)*. We further found that through heightened unethical request perceptions, the deceptive (vs. transparent) use of chatbots led to higher turnover intentions (indirect effect = 1.14, 95% CI = [0.551, 1.726]) and the decision to quit (indirect effect = 1.16, 95% CI = [0.656, 1.670]).

**Study 3**

The Hausman test was significant, (χ2(1) = 9.21, *p* = .002), showing that there might be an omitted variable bias, and that it is worthwhile to re-estimate the effect of trust on decision to hire where we used our manipulations as the instruments. The 2SLS analyses showed that trustworthiness had a significant positive effect on the decision to hire (b = 1.38, SE = 0.16, p < .001). Further, we also found a significant indirect effect of affiliation to transparent (vs. deceptive) chatbot use on decision to hire through increased levels of trust (indirect effect = 1.23, 95% CI = [0.448, 2.015]).

**Study 4**

The Hausman test was significant, (χ2(1) = 13.46, *p* < .001), showing that there might be an omitted variable bias. Using our experimental manipulation as the instrumental variables of trust, we found a significant effect of trust on decision to hire (*b =* 1.72, *SE* = 0.24, p <.001). Further, we also found a significant indirect effect of affiliation to transparent (vs. deceptive) chatbot use on decision to hire through increased levels of trust (indirect effect = 1.95, 95% CI = [0.788, 3.12])

**APPENDIX B**


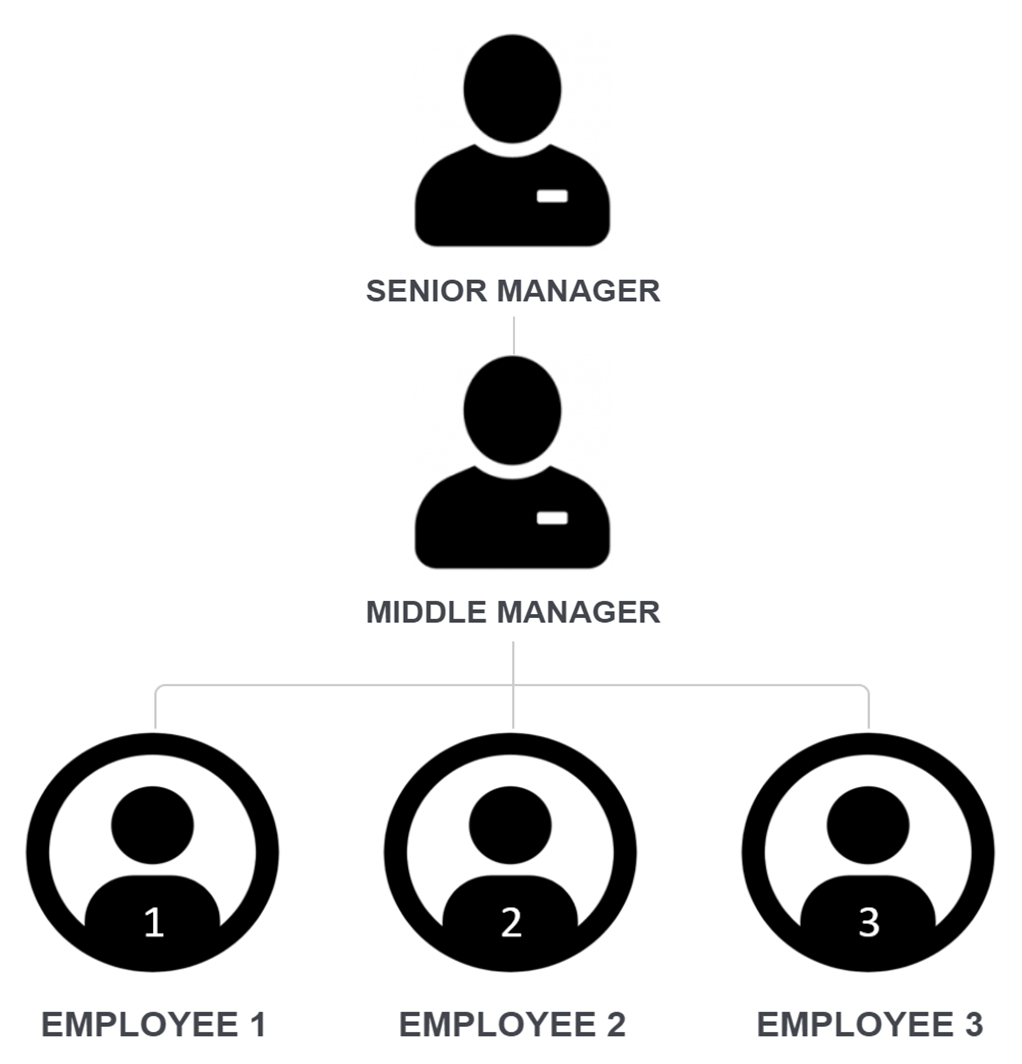


The image above illustrates the hierarchical representation of the simulated organization that participants were recruited to work for. All participants were allocated the position of “Employee 1” on what appeared to be a random basis.

**APPENDIX C**


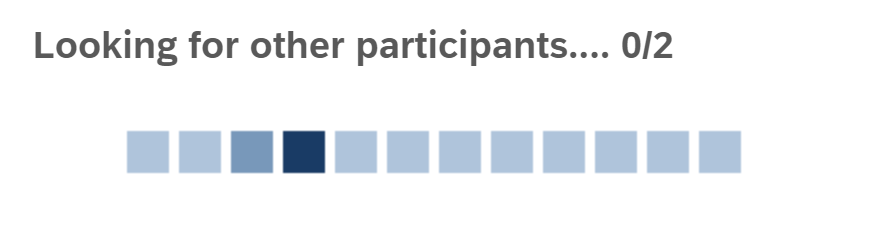

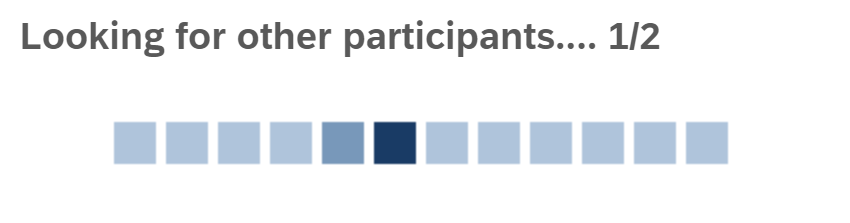

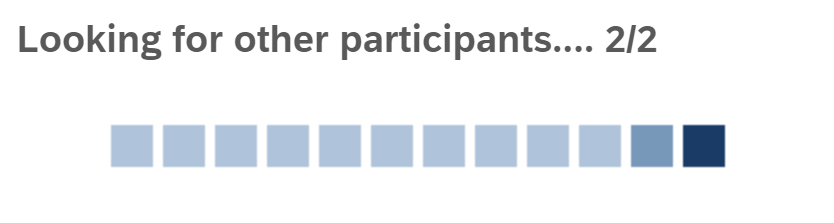


The imagine above illustrates the series of loading bars that appeared on the screen for participants. The loading bar was a gif-image where the dark blue square looped from left to right. After several seconds, the subsequent screen appeared so that participants saw that the counter (e.g., 1/2) went up. This animated progress bar was used to make participants believe that the system is busy connecting them, and that they had to wait until the system was ready.
